# Love under lockdown: How changes in time with partner impacted stress and relationship outcomes during the COVID-19 pandemic

**DOI:** 10.1177/02654075231162599

**Published:** 2023-03-09

**Authors:** Kaitlin Derbyshire, Sabrina Thai, Claire Midgley, Penelope Lockwood

**Affiliations:** 1 7938 University of Toronto, Toronto, ON, Canada; 2 7497 Brock University, Saint Catharines, ON, Canada; 3 2129 University of Calgary, Calgary, AL, Canada

**Keywords:** Romantic relationships, COVID-19, stress, satisfaction, commitment

## Abstract

With the onset of COVID-19, governments around much of the world implemented strict social distancing and stay-at-home orders that profoundly affected the amount of time many couples were spending together. In the present research, we examined whether perceptions of a change in time spent with a partner were associated with stress, and whether stress levels in turn predicted relationship commitment and satisfaction, both in the short term (Time 1) and longer term (Time 2; i.e., after 10 months). Results indicated partial mediation, such that less (vs. more) time spent with the partner was associated with greater stress at Time 1, which in turn partly accounted for lower commitment and relationship satisfaction both at Time 1, and satisfaction at Time 2. Less (vs. more) time spent with partner at Time 1 also predicted a greater likelihood of relationship dissolution at Time 2, again partially mediated by stress. An increase in quality time spent together at Time 2 predicted stress and relationship outcomes over and above the change in time spent together more generally. This research has important implications for understanding the ongoing effects of the pandemic on romantic relationships. In addition, this study provides new evidence regarding how changes in time spent with a partner are associated with stress and subsequent relationship outcomes.

## Introduction

The COVID-19 pandemic caused widespread uncertainty and distress for the world’s population on an unprecedented scale. Indeed, individuals in many countries reported experiencing increases in anxiety, stress, and depression. For example, in one study involving data primarily from the United States and Israel, a sizeable proportion (22.2%) of participants met the threshold for generalized anxiety during the pandemic, with anxiety scores highest among participants who were worried about COVID-19 (Barzilay et al., 2020). Another study focused on Canadian participants found that 38.2% of respondents in May 2020 reported a deterioration in their mental health as a result of the pandemic (Jenkins et al., 2021). Further, a review of the literature on COVID-related psychological distress found that, across a variety of studies in several different countries, participants reported moderately high symptoms such as anxiety (ranging from 6.33%-50.9% across studies), depression (ranging from 14.6%-48.3%), and stress (ranging from 8.1%-81.9%; Xiong et al., 2020). This pandemic-related distress in turn posed a significant threat to the well-being of romantic relationships (Pietromonaco & Overall, 2021). Indeed, a growing number of studies suggest that the pandemic has negatively impacted relationships (for a review, see Wagner et al., 2020), leading to increased relationship conflict (e.g., Luetke et al., 2020), reduced relationship satisfaction and commitment (e.g., Balzarini et al., 2022), and higher divorce rates (Kofman & Garfin, 2020).

One key pandemic-related change that may have influenced relationship outcomes is the shift in the number of hours that individuals were spending with their partners. With the onset of COVID-19, governments around the world implemented strict social distancing and stay-at-home orders to lessen the spread of the virus (United Nations, 2020); these measures profoundly affected the amount of time many couples were spending together. For some, including those who were not already living with their partner, restrictions in travel and social gatherings may have made it difficult for them to spend time with one another (Walsh, 2020). Whereas they have been able to see each other regularly before the pandemic, quarantines and lockdowns may have led to severe cutbacks in the time couples could spend together. For others, stay-at-home orders resulted in both partners working from home and eschewing in-person social activities with friends; thus, partners may have been together in the same living space without the breaks they would usually experience due to work or daily errands (Carlson et al., 2020).

### A change in time spent together impacts stress

In the present research, we examine how these changes in time spent with one’s romantic partner during the pandemic may have affected stress, and thus contributed to subsequent relationship outcomes. Our predictions were guided by attachment theory (Bowlby, 1969/1982; Hazan & Shaver, 1987; Simpson & Rholes, 2017), which highlights the benefits of proximity to, and costs of separation from, one’s primary attachment figure, particularly during distressing events. According to attachment theory, infants are motivated to form strong bonds with their primary caregivers to protect themselves from danger (Bowlby, 1969/1982). Indeed, infants’ internal control system functions according to the principles of physiological homeostasis in which maintaining close proximity, and thus accessibility, to a primary caregiver allows infants to maintain a deactivated inner control system that is in equilibrium; when infants’ security is threatened, their attachment system is activated, and they seek protection and comfort from an attachment figure in attempt to restore equilibrium (Bowlby, 1969/1982). Although attachment research was initially focused on children, the theory expanded to include adults’ bonds with their primary attachment figures, and their relationship partners in particular (Hazan & Shaver, 1987). Adults view their relationship partners as a “safe haven” (Doherty & Feeney, 2004) and thus seek contact with their partner during times of distress; the comfort and support provided by adults’ relationship partners, in turn, help to reduce their distress and restore their inner control system’s equilibrium (Hazan & Shaver, 1987; Simpson & Rholes, 2017).

Indeed, a number of studies suggest that the presence of a relationship partner can alleviate stress associated with threatening contexts. Individuals who are in physical proximity to a close other may perceive a threat as less demanding, and expect to have more resources to cope with that threat, resulting in lower stress (Beckes & Coan, 2011). Having accessible social support from a partner, and feeling confident that one’s partner will be available in times of need, serves a stress-buffering function, reducing the distress associated with adverse life events (Cutrona & Russell, 2017; Falconier et al., 2013; Feeney & Collins, 2019; Hilpert et al., 2018; Simpson & Overall, 2014). To the extent that partners provide supportive behaviors during threatening experiences, individuals experience a more rapid reduction in stress (Meuwly et al., 2012). Moreover, the presence of a romantic partner may provide benefits through physical contact; past research suggests that a partner’s touch may play an important role in reducing stress-related cortisol levels (Berretz et al., 2022) as well as self-reported stress (Jakubiak & Feeney, 2019). Given their status as providers of a “safe haven” (Doherty & Feeney, 2004), romantic partners may have played an especially important role for individuals coping with the threat posed by the pandemic.

Whereas the presence of the primary attachment figure may reduce stress, the absence of this figure is associated with heightened stress. Studies examining partners’ retrospective accounts of their separations or assessing the impact of long-term separations brought about by military deployment and job-related absences suggest that individuals typically report experiencing negative outcomes when separated from their partner (e.g., Gerstel & Gross, 1984; Hughes & Galinsky, 1994; Hughes et al., 1992; Medway et al., 1995; Riggs, 1990; Roehling & Bultman, 2002). More extended separations can be associated with anxiety, sleeplessness, anger, depression, and agitation along with other forms of behavioural and psychological dysregulation (e.g., Diamond et al., 2008; for a review, see Vormbrock, 1993). In general, loss of time with a romantic partner appears to elevate stress.

In sum, when faced with the threat posed by the COVID-19 pandemic, time spent with the partner may have had significant implications for individuals’ stress levels. To the extent that individuals’ security was threatened, the partner may have served as a refuge, a source of comfort and support (Shaver & Mikulincer, 2007). Individuals spending more time than usual with their partners may thus have experienced less stress, drawing benefits from their physical proximity to the close other (Beckes & Coan, 2011). In contrast, individuals spending decreased time with their partner may have experienced greater stress. Denied the comforting presence of the partner during this time of uncertainty and distress, individuals may have found the relative reduction in time with the partner to be particularly stressful.

We note that the benefits of additional time together may depend on individuals’ attachment styles (Mikulincer & Shaver, 2007). Anxiously attached individuals have a high need for proximity to others, especially their romantic partner (Goldbart & Wallin, 1997). Consequently, spending more time with their partner may have helped them to satiate this need. Avoidantly attached individuals, in contrast, tend to have negative views of their partner (Bartholomew, 1990; Simpson & Rholes, 2017) as a result of past experiences with an attachment figure who was rejecting or unavailable (Mikulincer & Shaver, 2007); avoidant individuals have learned that closeness may be problematic and that they should avoid relying on others. Accordingly, avoidantly attached individuals may have been unlikely to benefit from spending more time with the partner during the pandemic. More securely attached individuals expect their partner to be responsive to their needs, and thus should be more likely to benefit from the partner’s presence. However, the majority of individuals report being securely attached (Hazan & Shaver, 1987), and most individuals identify their partner as their safe haven during times of threat (Doherty & Feeney, 2004); thus, although we did include a measure of attachment style as a potential moderator, we have focused primarily on the possibility that more (vs. less) time spent with their partner would be associated with lower stress for most individuals.

We also note that it is unclear whether a change in time together, or a change in *quality* time together, would be responsible for a change in stress, and subsequent relationship outcomes. That is, time with the partner may be most beneficial when that time is of a high quality, such that the partner is available to provide comfort and reassurance, and is not simply present in the same place at the same time. Indeed, past studies suggest that individuals are most likely to benefit from their partner’s presence when they perceive the partner to be actively providing supportive behaviors (e.g., Meuwly et al., 2012). Thus, we examined whether an increase in quality time, rather than the increase in time spent with the partner more generally, might be important in predicting stress.^1^

### Change in time spent together and stress: Alternative models

**Figure 1.**
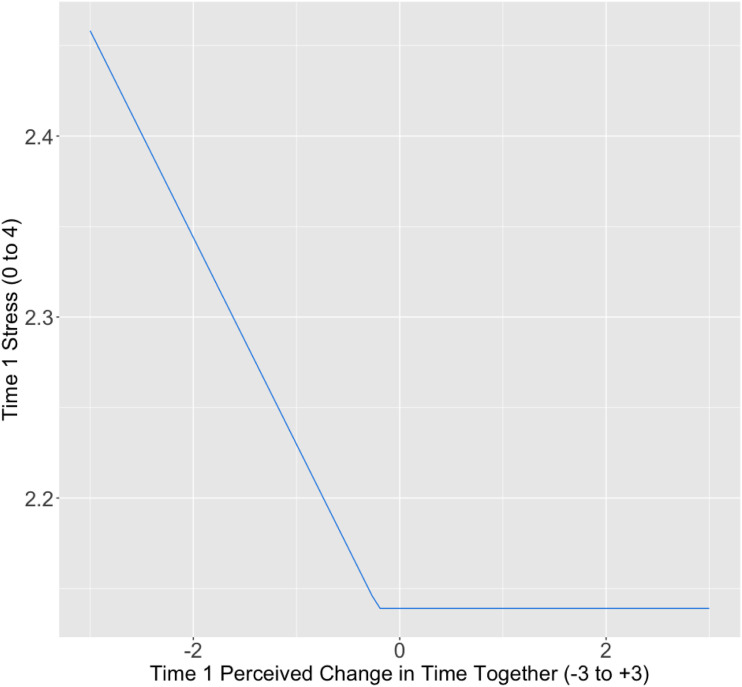
Linear Plateau Model for T1 Subjective Change in Time on T1 Stress.

Up to this point, we have discussed a linear relationship between changes in time together and stress, with more (vs. less) time together resulting in decreased stress. However, we also considered two alternative possibilities. First, it is possible that a change in time with the partner has the greatest impact on stress when individuals are spending *less* time with the partner than usual; under such circumstances, a smaller reduction in time together may be associated with lower stress. In contrast, increased time with the partner, over and above pre-pandemic levels, may yield limited returns, provided that individuals had sufficient time with their partners for their attachment needs before the pandemic started. This model, while still consistent with attachment theory, would suggest that increased time spent together yields diminishing returns, with less time together than usual associated with higher stress, but more time spent together than usual showing a “linear plateau” effect, such that additional time together conferred little advantage beyond the status quo (see Figure 1).

Second, we considered the possibility that increases in time together would be associated with reduced stress up to a point, beyond which the unprecedented increase in time with the partner may have increased stress. This would be consistent with anecdotal accounts suggesting that the pandemic served as a “pressure cooker,” in which couples who were trapped in their homes without breaks from each other may have suffered from the excessive amount of time spent together without other social contacts (e.g., Harris & Tarchak, 2020; Savage, 2020; Stroh, 2020). This possibility would suggest a curvilinear relationship between time spent together and stress. Our data enabled us to assess whether changes in time together would yield the predicted linear effect on stress, and also to assess two alternative relationships in which this effect either levelled off in a linear plateau, or was curvilinear.

### Stress and relationship outcomes

A significant body of research has demonstrated that individuals’ stress levels can affect their commitment to and satisfaction with their relationship (Karney and Bradbury, 1995). Accordingly, in addition to examining how time spent together would be associated with stress, we also assessed how the stress associated with changes in time spent with the partner may in turn have affected relationship outcomes. Past research suggests that an increase in stress is associated with more negative cognitions about the partner and relationship (Neff & Karney, 2004; Tesser & Beach, 1998), more problematic relationship-related behaviors (Bolger et al., 1989; Williamson et al., 2013), and less satisfying interactions with the partner (Repetti, 1989; Turliuc & Campbell, 2021). Further, longitudinal studies suggest that stress leads to lower relationship quality and greater relationship discord over time (Bodenmann, 1997; Neff & Karney, 2017). Thus, we expected that increased levels of stress associated with changes in time spent with the partner would predict reduced relationship satisfaction and commitment and greater likelihood of relationship dissolution.

### Relationship outcomes during the COVID-19 pandemic

To date, research has not examined how changes in the amount of time couples spent together as a result of the pandemic may have impacted stress and subsequent relationship outcomes. A number of studies have provided evidence suggesting that the stress associated with the pandemic has had a negative impact on relationships (e.g., Luetke et al., 2020; Pieh et al., 2020; Pollard & Rogge, 2022), including lower levels of relationship satisfaction and commitment (Balzarini et al., 2022), increased intentions to terminate relationships (Li & Samp, 2021), increased relationship instability (Ogan et al., 2021), and high rates of contacting alternative sexual and romantic partners (Lehmiller et al., 2020). Although these studies provide important evidence regarding the role of stress in pandemic-related negative relationship outcomes, it remains unclear whether the change in time spent together may itself have contributed to these problems. Two studies that examined couples’ functioning during early lockdowns found that individuals quarantining with their partners, and thus presumably experiencing an increase in time together, reported positive rather than negative relationship outcomes. Specifically, in one study of cohabiting couples in Spain, participants reported relational improvement rather than deterioration during the first three weeks of state-regulated lockdown (Günther-Bel et al., 2020). Another study of couples under lockdown in Belgium found that relationship satisfaction increased between March and July 2020 (Galdiolo et al., 2022). These studies provide important evidence suggesting that the presence of the partner during the pandemic was beneficial. They did not, however, examine how the relative change in time was affecting stress, and focused only on those couples living together; thus, it remains unclear how the full range of changes in time (both more and less) with a partner may have affected individuals. In one study that did explicitly assess time spent with a partner during the pandemic, a decrease in shared time was associated with a perceived decrease in relationship satisfaction (Vigl et al., 2022); however, this research did not examine the role of stress in this association, nor the impact of changes in shared time together on relationship outcomes over time.

### The present research

We collected our first wave of data in February 2021 (Time 1), when the Alpha variant was surging in a massive wave of new cases in Europe and North America, and South Africa was grappling with the Gamma variant (CBC, 2021; Lew et al., 2022). Lockdown and social distancing measures that had been relaxed the previous autumn (e.g., Lew et al., 2022; The New York Times, 2022) were reintroduced. We conceptualized these events as a stressor that was experienced by all participants in our sample. We then investigated whether individuals’ perceptions of the change in the amount of time spent with a relationship partner during this timeframe would predict stress, and whether stress would in turn predict relationship commitment and satisfaction.^2^

In addition, we considered whether time with friends or family may have moderated the effect of time together on stress. Individuals have attachment networks beyond their romantic relationships, including parents, siblings, and friends (Doherty & Feeney, 2004); these individuals may also provide some benefits as a safe haven in times of distress. Accordingly, we examined whether the benefits of a partner’s presence (and costs of a partner’s absence) may have been greatest for individuals isolated from friends and family. Moreover, because time spent together may confer the greatest benefits on more securely attached individuals, we also considered attachment style as a possible moderator.

We collected a second wave of data in December 2021 (Time 2), roughly 10 months later. At this time, COVID-19 cases were once again rising, with the impact of the Omicron variant starting to be felt in many countries (or already raging, as in the UK; Elliot et al., 2022). By examining the same participants in this second wave, we were able to assess whether the stress associated with time spent together would have longer-term implications for satisfaction, commitment, and relationship dissolution. Moreover, measures included at Time 2 (T2) also enabled us to assess whether it was quality time together, or time together more generally, that predicted relationship outcomes.

We first tested whether a relative increase in time spent together during the uncertainty of the pandemic would predict a decrease in stress at Time 1 (T1). We then examined whether the stress associated with a change in time together would predict commitment and relationship satisfaction. Specifically, we used a mediation model to test whether a relative increase in time together would predict lower stress, which in turn would predict greater commitment and satisfaction. We next examined possible moderators of the association between a change in time together and stress. We expected this association to be strongest for participants who were a) spending less time with friends and family, and b) less avoidantly attached. Further, in addition to testing a linear relationship between change in time together and stress, we also tested two alternative models: one in which the relationship between the change in time spent together and stress plateaued, suggesting that additional time with the partner may yield diminishing returns; and one in which the relationship between the change in time spent together and stress was curvilinear, suggesting that either a decrease or increase in time together might be stressful.

We also examined whether these relationships among a change in time together, stress, and relationship outcomes would hold over time: We assessed whether the stress associated with time together at T1 would predict relationship commitment, satisfaction, and relationship dissolution 10 months later (T2). Finally, we tested whether an increase in quality time, rather than the increase in time spent with the partner more generally, would be important in predicting stress and, subsequently, relationship outcomes at T2.

In sum, the present research provides new insights into how the pandemic has affected relationships, examining how the change in time together may have impacted stress and subsequent relationship outcomes. This study also has broader implications for the literature on relationships more generally: Whereas past studies have primarily focused on the negative effects of physical separations on relationships, our research examines how a relative change in time spent together may be associated with linear, plateauing, or curvilinear changes to stress levels, with subsequent impacts on relationship outcomes. Finally, by showing that these effects may have implications over time, we demonstrate how the pandemic, through changing the degree to which individuals’ partners were present or absent in their day-to-day lives, may have had longer-term effects on relationships.

## Method

### Participants

Participants (*N* = 711) were recruited through Prolific for an online study on romantic relationships. Participants were eligible to take part if they indicated in a pre-screening questionnaire that they had been in an exclusive relationship for at least 12 months. Participants were compensated at a rate of £0.125 per minute of completing the questionnaire. We excluded 141 participants at T1: 25 did not complete the survey, 25 were not in an exclusive romantic relationship, four were in their current relationship for less than 12 months, and 93 failed one or more of four attention checks (in some cases, participants were excluded for more than one reason).^3^ In total, we had usable data from 570 participants (272 female, 293 male, 5 nonbinary; *M*_age_ = 27.71, *SD* = 8.67, range = 18 to 69, *Mdn* = 25.00; *M*_relationship length_ = 72.98 months, *SD* = 74.35, range = 12 to 495, *Mdn* = 48.00). Of these participants, 464 identified their sexual orientation as heterosexual, 17 as gay or lesbian, 52 as bisexual, 8 as queer, 8 as questioning, 3 chose not to answer, and 8 chose other (e.g., asexual, pansexual). The majority of our participants resided in Europe (87.37%) or North America (10.70%). For ethnicity, 484 participants identified as White, 10 as Black, 21 as South American, 4 as Middle Eastern, 5 as South Asian, 4 as Southeast Asian, 3 as East Asian, 33 as other, and 9 as multiracial. One hundred and eighteen reported that they had completed high school, 62 had completed some college, 122 had completed some university, 8 had completed an Associate degree, 165 had completed a Bachelor’s degree, and 95 had completed a Postgraduate degree. One hundred and thirty-eight (24.21%) reported that there were children living in the household, and 309 (54.21%) had been cohabiting with their partner since the onset of the pandemic (i.e., staying in the same household for the majority of days each week together).

Of the 570 participants included in the analyses for T1, 417 completed the second survey. We excluded 39 participants who were single, widowed, or in a different relationship at T2, and 26 who did not pass an attention check. In total, our analyses included 359 participants who provided usable data at both time points (182 female, 174 male, 3 nonbinary; *M*_age_ = 28.77, *SD* = 8.85, range = 18 to 63; *Mdn* = 26.00; *M*_relationship length_ = 79.42 months, *SD* = 77.12, range = 12 to 495, *Mdn* = 50.00). Our dataset examining relationship status as an outcome included 389 participants (201 female, 185 male, 3 nonbinary; *M*_age_ = 28.30, *SD* = 8.71, range = 18 to 63, *Mdn* = 25.00).^4^ Participants who completed our T2 survey did not differ in perceived change in time spent together, stress, commitment, or satisfaction, from those who did not complete our T2 survey, *t*s < 1.59, *p*s > .112.

### Procedure

Participants first completed a set of measures regarding their relationship, including their relationship status, whether or not they were living together, whether or not they had children living with them, as well as demographic information. Next, participants rated their perceptions of the change in time spent with their partner since the onset of COVID-19 on four items (“Since the start of the pandemic, I am spending _____ time with my partner than before the pandemic started;” “Since the start of the pandemic, my partner and I are at home together for _____ hours than before the pandemic;” “I am ______ often in the same room as my partner (other than during sleeping hours) now vs. before the pandemic;” “I spend _____ hours interacting with my partner now vs. before the pandemic.”). Ratings were made on a 7-point scale ranging from −3 (*much less/many fewer/much less/many fewer*) to +3 (*much more/many more/much more/many more*), with a score of zero indicating no change in the amount of time spent together. Ratings were averaged to form a single index of perceived change in time spent with partner (α = .92).^5^

In addition, participants completed two single item measures assessing change in time spent with friends (“Since the start of the pandemic, I spend ______ hours with my friends than before the pandemic started”) and change in time spent with family (“Since the start of the pandemic, I spend _______ hours with my family than before the pandemic started”). Ratings were made on a 7-point scale with endpoints ranging from *–3* (*many fewer*) to +*3* (*many more*).

Participants next completed the 36-item Experiences in Close Relationships-Revised (ECR-R) Adult Attachment Questionnaire (Fraley et al., 2000). The ECR-R includes items assessing two dimensions: 18 items are related to attachment-related anxiety (e.g., “I often worry that my partner will not want to stay with me*”*), and 18 items are related to attachment-related avoidance (e.g., “I get uncomfortable when a romantic partner wants to be very close*”*). Ratings were made on a 7-point scale (0 = *Strongly disagree*; 6 = *Strongly agree*)*.* Ratings were averaged separately for each subscale to produce a mean for both attachment anxiety (α = .93) and attachment avoidance (α = .93), after first reverse-scoring negatively-framed items.

Participants then completed a 10-item measure assessing their level of stress (Cohen et al., 1983) by indicating the extent to which they found situations in their life stressful (e.g., *“*In the last month, how often have you found that you could not cope with all the things that you had to do?”; α_T1_ = .88; α_T2_ = .89) on a 5-point scale (0 = *never*, 4 = *very often)*. They also completed a 7-item measure of their commitment to the relationship (e.g., “I am committed to maintaining my relationship with my partner”; Rusbult et al., 1998) on a 7-point scale (0 = *don’t agree at all*, 6 = *agree completely*; α_T1_ = .92; α_T2_ = .89). In addition, they completed a 5-item measure of relationship satisfaction (e.g., “I feel satisfied with our relationship”; Rusbult et al., 1998) using the same scale (α_T1_ = .93; α_T2_ = .93). Several other measures not relevant to the present hypotheses were also collected and are not discussed further.

#### T2 measures

After 10 months, participants were invited to complete a follow-up questionnaire in which they again completed a single-item measure of change in time spent with partner (“I currently spend _____ time with my partner compared to before the pandemic started”). They also completed single-item measures regarding the change in time with partner since T1 (“I currently spend _____ time with my partner compared to 10 months ago when I filled out this questionnaire [i.e., February 2021]),” as well as the change in quality time spent with partner since T1 (“I currently spend _____ quality time with my partner than when I last completed this questionnaire 10 months ago [i.e., February 2021]).” Ratings were made on a 7-point scale (−3 = *much less*, +3 = *much more*). In addition, participants completed the same measures of stress, commitment, and satisfaction as they had at T1. Finally, participants indicated whether they were still in the same romantic relationship as they had been at T1.

## Results

### Overview of results

We first ran a series of analyses to test our key hypotheses regarding the change in time together and stress, and stress as a possible mediator of the association between the change in time together and relationship outcomes at T1. We then tested possible moderators of the association between the change in time together and stress at T1. In addition, we examined alternatives to the linear relationship between the change in time together and stress at T1. Next, we ran analyses to test our key hypotheses at T2; that is, we assessed whether the change in time together at T1 would be associated with stress at T1, and whether this would in turn predict relationship outcomes at T2. Finally, we examined whether a change in time together, or a change in *quality* time together, would predict relationship outcomes at T2.

Descriptive statistics for all key variables at T1 and T2 are presented in Table 1. Correlations among key variables at both time points are reported in Table 2.

**Table 1.** Descriptive Data for Key Variables.

	Participants with data at T1 (*n* = 570)		Participants with data at T1 and T2 (*n* = 359)						
	T1		T1		T2		*t*	*p*	*d*
Variable	*M*	*SD*	*M*	*SD*	*M*	*SD*			
Perceived Change in Time Spent with Partner (+3 to −3)	0.28	1.70	0.22	2.01	0.18	1.68	0.33	.742	.02
Attachment anxiety (0 to 6)	1.97	1.26							
Attachment avoidance (0 to 6)	1.56	1.02							
Commitment (0 to 6)	5.20	1.05	5.32	0.89	5.21	0.92	2.72	.007	.14
Satisfaction (0 to 6)	4.50	1.29	4.59	1.23	4.52	1.17	1.31	.192	.07
Stress (0 to 4)	2.22	0.81	2.22	0.79	2.03	0.79	4.58	<.001	.24

*Note. t*’s represent paired *t-*tests and *d*’s are effect size estimates using Cohen’s *d*. To ensure a more concise follow-up questionnaire, participants at Time 2 completed only one of the four items assessing a change in time spent together since the start of the pandemic (“I currently spent ___ time with my partner compared to before the pandemic started”). For comparison purposes, the *M* and *SD* for this item only, rather than the mean of the four-item composite, is provided here for Time 1. Attachment anxiety and avoidance were measured at Time 1 only.

**Table 2.** Summary of correlations among key variables at Time 1 and Time 2.

	*N*	1	2	3	4	5	6	7	8	9	10	11	12
1. T1 Change in Time Spent with Partner	570	--											
2. T1 Stress	570	−.11*	--										
3. T1 Commitment	570	.17**	−.13**	--									
4. T1Satisfaction	570	.16**	−.28**	.68**	--								
5. T1Attachment Anxiety	570	−.12**	.45**	−.18**	−.43**	--							
6. T1Attachment Avoidance	570	−.11*	.25**	−.58**	−.66**	.39**	--						
7. T1 Change in Time Spent with Friends	570	.12**	−.05	−.08	.00	.01	.04	--					
8. T1 Change in Time Spent with Family	569	−.33**	.03	−.13**	.00	.07	.06	.22**	--				
9. T2 Change inTime Spent with Partner	359	−.13*	−.03	.06	.07	−.07	−.03	.01	.08	--			
10. T2 Change in Quality Time	359	.00	−.09	.04	.09	−.12*	−.09	.05	−.02	.59**	--		
11. T2 Commitment	359	.04	−.04	.65**	.46**	−.13*	−.43**	−.09	−.08	.08	.15*	--	
12. T2 Satisfaction	359	.04	−.19**	.48**	.65**	−.30**	−.47**	.04	−.01	.14*	.30**	.61**	--

*Note.* For correlations involving T1 variables only, we used all the usable data available at T1. For correlations involving T2 variables, we included usable data from T2 only. *p < .05. **p < .01.

### T1 analyses

#### Change in time spent together

A one sample *t*-test revealed that participants reported that they were spending more time with their partner than before the pandemic (i.e., the average score was significantly above the “0” score indicating no change in time spent together), *t*(569) = 3.99, *p* < .001, *r* = .16. This effect size suggests that the difference between the sample mean (*M* = 0.28, *SD* = 1.70) and the midpoint of the scale, zero, was a small-to-medium effect. Overall, 34.74% of participants reported less time, 10.70% reported no difference, and 54.56% reported spending more time with their partners than pre-pandemic.

#### Change in time with friends and family

A one-sample t-test revealed the participants reported that they were spending less time with their friends (*M* = −1.98, *SD* = 1.28)*, t*(569) = -36.91, *p* < .001, *r* = .84, and less time with family (*M* = −0.56, *SD* = 1.99), *t(*568) = −6.69, *p* < .001, *r* = .27, than before the pandemic (i.e., average score significantly below “0”).

#### Attachment

As reported in Table 1, participants’ scores on both the attachment avoidance subscale and the attachment anxiety subscale were relatively low, indicating that participants in the sample were generally characterized by more secure attachments. Indeed, our sample reported lower scores on both the anxiety and avoidance subscales than a normed sample (Fraley, 2013); one-sample *t*-tests for anxiety, *t*(569) = -11.17, *p* < .001, *r* = .42, and avoidance, *t*(569) = -8.33, *p* < .001, *r* = .33, confirmed that our means were significantly lower than data from this normed sample.

#### Stress

Perceived stress in the sample was relatively high (see Table 1 for means). This is perhaps not surprising, given that data was collected when the pandemic was posing a very considerable threat worldwide. To test the possibility of a linear relationship between a change in time together and stress, we regressed perceived stress on perception of time together. As predicted, participants who perceived that they were spending relatively more (vs. less) time with their partner reported lower levels of stress, *b* = −0.05, *SE =* 0.02, *t*(568) = -2.74, *p* = .006, *r* = .11.^6^

#### Mediation analyses (T1)

**Figure 2.**
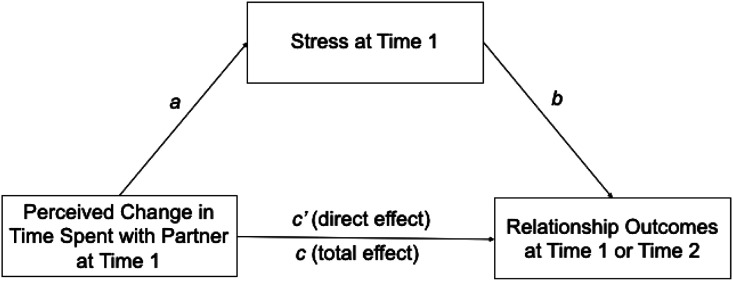
Conceptual Model of Mediation.

Next, using the PROCESS macro and bootstrapping procedure (Hayes, 2018), we conducted a set of mediation analyses to examine whether stress mediated the association between a change in time spent together and relationship outcomes (commitment and relationship satisfaction) using a random seed of 30,217 (see Figure 2). We used 5000 resamples to provide stable estimates of the direct, indirect, and total effects. 95% confidence intervals were determined from the bootstrap resamples, and any interval that did not include zero was considered to be significantly different from zero. All mediation results are reported in Table 3.^7^

**Table 3.** Summary of mediation analyses testing whether perceived change in time spent together predicts stress at T1, which in turn statistically accounts for relationship outcomes reported at T1 and T2 (10 months later).

	Mediation statistics				
Outcome	T1 Perceived Change in Time → T1 Stress (*a* path)	T1 Stress → Outcome (*b* path)	T1 Perceived Change in Time → Outcome (*c’* path)	Indirect Effect (*ab*)	Total Effect (*c* path)
T1 Commitment					
	*b* = −0.05 (0.02) [-0.09, −0.02] *p* = .006**	*b* = −0.15 (0.05) [-0.25, −0.04] *p =* .006**	*b* = 0.10 (0.03) [0.05, 0.15] *p* < .001***	*b* = 0.01 (0.004) [0.001, 0.02]	*b* = 0.11 (0.03) [0.05, 0.16] *p* < .001***
T1 Satisfaction					
	*b* = −0.05 (0.02) [-0.09, −0.02] *p* = .006**	*b* = −0.42 (0.06) [-0.55, −0.30] *p <* .001***	*b* = 0.10 (0.03) [0.04, 0.16] *p* = .002**	*b* = 0.02 (0.01) [0.01, 0.04]	*b* = 0.12 (0.03) [0.06, 0.18] *p* < .001***
T2 Commitment					
	*b* = −0.06 (0.03) [-0.11, −0.01] *p* = .014*	*b* = −0.04 (0.06) [-0.16, 0.08] *p = .*548	*b* = 0.02 (0.03) [-0.04, 0.08] *p =* .562	*b* = 0.002 (0.004) [-0.006, 0.01]	*b* = 0.02 (0.03) [-0.04, 0.08] *p =* .507
T2 Satisfaction					
	*b* = −0.06 (0.03) [-0.11, −0.01] *p* = .014*	*b* = −0.28 (0.08) [-0.43, −0.13] *p* < .001***	*b* = 0.01 (0.04) [-0.07, 0.08] *p* = .818	*b* = 0.02 (0.01) [0.003, 0.04]	*b* = −0.03 (0.04) [-0.05, 0.10] *p =* .487
T2 Relationship Status					
	*b* = −0.08 (0.02) [-0.12, −0.03] *p* = .002*	*b* = 0.64 (0.27) [0.11, 1.16] *p* = .018*	*b* = −0.46 (0.12) [-0.70, −0.22] *p* < .001***	*b* = −0.05 (0.03) [-0.12, −0.005]	*—*
					*—*

*Note.* Relationship status was a binary variable such that 0 = *the same relationship* and 1 = *broken up*. Values reported in parentheses represent standard errors. Values reported in square brackets represent bootstrapped 95% confidence intervals. Total effects are unavailable with dichotomous outcome.

The total effect of the change in time spent together on commitment was significant. That is, participants who perceived that they were spending less time with their partner reported lower commitment. In addition, the indirect effect of a change in time spent together on commitment was significant. That is, participants who spent less (vs. more) time together during the pandemic than before reported more stress, which in turn predicted lower commitment at T1. The direct effect of a change in time on commitment, however, was still significant when the indirect path through T1 stress was taken into account. Thus, participants who spent less time with their partner during the pandemic experienced a decline in commitment in part because they felt more stressed.

The total effect of the change in time spent together on satisfaction was significant. That is, participants who perceived that they were spending less time with their partner reported lower satisfaction. The indirect effect of a change in time together on relationship satisfaction was significant. That is, participants who spent less (vs. more) time together during the pandemic than before reported more stress, which in turn predicted less satisfaction at T1. The direct effect of a change in time together on satisfaction remained significant when the indirect path through T1 stress was taken into account. Thus, participants who spent less time with their partner during the pandemic experienced a decline in satisfaction in part because they felt more stressed.^8^

#### Moderation analyses

We then examined, in two separate models, whether the effect of time together on stress would be moderated by two variables, a change in time with family and a change in time with friends. In each model, we entered the effects of time together and the moderator, and the interaction between time together and the moderator. For significant interactions, we examined the simple effect of the moderator at 1 *SD* above and below the midpoint of time together with partner (Aiken & West, 1991), which was meaningfully labelled as no change in time.

Change in time with family did not moderate the effect of time together with partner, *b =* -0.01, *SE* = 0.01, *t*(565) = -1.30, *p* = .194, *r* = .05. Our second moderation analysis revealed a significant interaction between time spent with partner and time with friends, *b* = 0.05, *SE* = 0.02, *t*(566) = 3.19, *p* = .001, *r* = .13. For participants who were spending less time than usual with their partner, more time with friends was associated with less stress, *b* = −0.15, *SE* = 0.05, *t*(566) = -3.12, *p =* .002, *r* = .13. For participants who were spending more time than usual with their partner, however, there was no effect of time with friends on stress, *b* = 0.04, *SE* = 0.03, *t*(566) = 1.19, *p* = .234, *r* = .05. Thus, individuals benefited from the effects of extra time with friends, but only when they were spending less time than usual with their partners.

Next, we examined the possibility that attachment anxiety and attachment avoidance moderated the association between time spent together and stress at Time 1. In this model, we entered time together, attachment anxiety, attachment avoidance, the anxiety by time together interaction, and the avoidance by time together interaction. The main effect of attachment anxiety, *b* = 0.33, *SE* = 0.03, *t*(564) = 10.17, *p <* .001, *r* = .39, and attachment avoidance, *b* = 0.07, *SE* = 0.03, *t*(564) = 2.11, *p =* .035, *r* = .09, were significant, such that individuals higher in anxiety or avoidance reported greater stress. Neither the anxiety by time nor the avoidance by time interactions were significant, *t*s < 0.76, *p*s > .446.

#### Alternatives to linear model

We next tested two alternatives to the linear model in which more (vs. less) time with the partner was associated with greater stress. First, we tested whether spending more time together with the partner, over and above pre-pandemic levels, may yield limited returns in reducing stress. We examined this possibility by fitting the data to a model with a linear-plateau; this is a segmented model in which part of the data is characterized by a linear trend and another part is characterized by a zero-slope plateau. Using the nlsLM function in the nrlaa package (Miguez, 2020) in R, we fit a self-starting linear plateau model to our data. This analysis revealed that as participants report smaller decreases in time together, stress levels decrease until participants report no change in time together, *b =* -0.11, *SE =* 0.07, *t*(567) = -1.72, *p* = .086, at which point stress levels plateaued when participants reported no change in time spent together, *b* = −0.21, *SE* = 1.28, *t*(567) = -0.16, *p* = .872,^9^ at a value of 2.14 on our stress measure. That is, the point at which the plateau begins in the model is not significantly different from the zero point on the time together scale, which was labelled no change in time. Stress decreases in a linear fashion as time together increases, but only up to the point at which participants report spending the same amount of time together as they did before the pandemic. Beyond this zero point, the association plateaus, such that any additional increase in time together did not predict a reduction in stress.

Next, we examined various fit indices to determine whether the linear-plateau model fit the data better than the linear model. The linear-plateau model (*R*^2^ = .02) explained more variance than the linear model (*R*^2^ = .01), had the same Root Mean Squared Error as the linear model (RMSE = 0.80), and a lower AICc (1365) than the linear model (1368.95). Thus, although the linear model fits the data, the linear-plateau model provides a modest improvement in fit. Overall, this analysis suggests that for participants who were spending less time than usual with the partner, a smaller reduction in time was associated with less stress; however, spending more time with the partner than usual during the pandemic yields limited returns in reducing stress. Once participants’ stress levels were 0.10 *SD* below the sample mean for stress, they no longer reaped any benefit from additional time with the partner (see Figure 1).

Second, we considered whether time together might be associated with reduced stress up to a point, but that beyond this point, an increase in time with the partner may have increased stress; we tested this possibility by fitting a curvilinear model to the data. In this model, we regressed stress on time together and time together squared. There was no significant effect of the squared effect of time together, *b* = 0.02, *SE* = 0.01, *t*(567) = 1.55, *p* = .123, *r* = .06. Thus, the curvilinear model did not provide a better representation for the relationship than did the linear model or linear-plateau model.

Overall, our findings suggest that individuals who were spending less time than usual with their partners were experiencing greater stress. Those spending more (vs. less) time with their partners were experiencing less stress, but only up to a point; once individuals were spending the same amount of time with their partner as they had before the pandemic, any additional time together did not lead to an extra reduction in stress.

### T2 analyses

Relative to T1, participants at T2 reported lower commitment and lower stress, but similar levels of relationship satisfaction (see Bartholomew, 1990Table 1).

#### Mediation analyses (T2)

We next conducted a set of analyses to examine whether the effects of a change in time spent together at T1 would continue to predict commitment and satisfaction at T2, and whether this association was mediated by stress level at T1. That is, we examined whether participants who were spending less time than usual with their partner early in the pandemic would experience greater stress, and whether this in turn would be associated with greater relationship commitment and satisfaction 10 months later. We also examined whether a change in time spent together at T1 would predict relationship dissolution at T2, and whether this would also be mediated by T1 stress. Full results are reported in Table 3.

The indirect effect of T1 time spent together on commitment at T2, mediated by stress at T1, was not significant. The indirect effect of a change in time spent together at T1 on relationship satisfaction at T2 was significant. That is, participants who spent less (vs. more) time together during the pandemic than before reported more stress at T1, which in turn predicted less satisfaction at T2. Thus, participants who spent less time with their partner earlier in the pandemic experienced a decline in satisfaction later in the pandemic because they felt more stressed. The total and direct effects were not significant.

The indirect effects of a change in time together on relationship status at T2 was significant. That is, participants who spent less (vs. more) time together during the pandemic than before reported more stress, which was associated with a greater likelihood of having broken up at T2. The direct effect of a change in time on relationship status, however, was still significant when the indirect path through T1 stress was taken into account. Thus, participants who spent less (vs. more) time with their partner earlier in the pandemic were more likely to break up later in the pandemic in part due to the higher stress they had experienced at T1.

#### Quality of time spent together (T2)

Our data at T2 also enabled us to consider another aspect of time spent together: the quality of that time. Whereas some participants may have been spending more time together simply because they were sharing home office space, others may have been spending more time actually interacting with their partner, and thus have experienced more “quality” time together. At T2, we had measured both the increase in time spent together as well as the increase in quality time spent together since T1. We regressed each T2 outcome onto T2 quality of time and T2 perception of time together, relative to T1. An increase in quality time spent with partner was negatively associated with stress, *b* = −0.17, *SE =* 0.04, *t*(356) = -4.41, *p* < .001, *r* = .23, and positively associated with commitment, *b* = 0.11, *SE =* 0.05, *t*(356) = 2.31, *p* = .021, *r* = .12, and satisfaction, *b* = 0.30, *SE =* 0.06, *t*(356) = 5.26, *p* < .001, *r* = .27, controlling for the general increase in time spent with partner since T1. The effect of time spent together at T2, relative to T1, on stress, commitment, and satisfaction, was no longer significant once quality of time was included in the model, *t*s < 1.50, *p*s > .135. Thus, spending additional quality time with one’s partner appears to play an especially important role in reducing stress and enhancing relationship outcomes, over and above an increase in time together more generally.

## Discussion

The COVID-19 pandemic created an extraordinary situation in which many individuals experienced a marked change in the amount of time they were spending with their partner. Whereas some saw less of each other due to lockdowns or possibly long work hours, others found themselves almost constantly in each other’s company, unable to leave the home to work or socialize. In the present study, we found that this change in the amount of time individuals were spending with their partner had significant implications for their relationship. Participants who reported spending less (vs. more) time with their partner during the pandemic experienced greater stress, which in part accounted for their lower commitment and relationship satisfaction.

The present research also highlights the persistence of these effects across time. Individuals who reported spending less (vs. more) time with their partner in February of 2021 experienced an increase in stress, which was associated with more negative outcomes for the relationship not only at that time but also with a continued drop in relationship satisfaction several months later, and with a greater likelihood that the relationship would end. We did not, however, find a long-term effect for commitment. It may be that these effects persisted, at least for satisfaction, because the initial effects of a reduction in time spent together at T1 had negative implications for the quality of partners’ interactions with each other, leading to an ongoing decrease in relationship quality. Additional analyses for the T2 data suggest that the positive effects of more (vs. less) time together on stress reduction and the subsequent benefits for relationship outcomes were more pronounced when that time was of higher quality.

Although the present research focuses on a change in time together as a result of a specific external event, a global pandemic, our findings have broader implications for theory and research on relationships. Couples may experience a change in time spent together for a variety of reasons; some are relatively commonplace, such as the birth of a child, or work-related obligations, and others are more dramatic, such as circumstances arising from war or natural disasters. Although past studies have examined separations, in which individuals are completely apart from the partner (for a review see Vormbrock, 1993), less is known about how individuals navigate changes that result, not in a total separation, but rather in a reduction or increase in time spent with their partner. We found that decreases in time together are potentially problematic, resulting in greater stress, which in turn contributes to drops in commitment and satisfaction, and even longer-term effects on the longevity of the relationship itself. According to the attachment framework (e.g., Hazan & Shaver, 1987), reduced access to the primary attachment figure may be a stressful experience: Individuals may experience distress when the attachment figure is less available to provide support and reassurance. During the pandemic, itself a major source of stress and uncertainty (Xiong et al., 2020), the reduction in time with the attachment figure may have been particularly upsetting. At a time when people were most in need of security and comfort, a loss of time with the person most likely to be perceived as a safe haven (Doherty & Feeney, 2004) was associated with increased stress. For those individuals spending extra time with their partner, relative to before the pandemic, the additional time conferred only limited benefits; to the extent that individuals already had sufficient access to their partner for reassurance and support, the additional time may have had little added value. In future research, it will be important to examine the circumstances under which a change in time spent together is most detrimental or beneficial, and whether this effect is equally strong when couples are not simultaneously experiencing a negative external event.

The importance of time spent with the partner may also vary depending on access to other key members of one’s social network. For example, we found that when individuals were spending less time than usual with their partner, extra time with friends was associated with reduced stress. When the primary attachment figure is unavailable, individuals may be able to take advantage of other interpersonal resources. Indeed, past research suggests that friends can serve as preferred attachment figures (Doherty & Feeney, 2004). We did not, however, find similar effects for time with family members.

Unexpectedly, the impact of time together on stress was not moderated by attachment style. We had predicted that less securely attached individuals, and avoidantly attached individuals in particular, may have benefitted less from the partner as a safe haven; given their negative working model of the partner, it would be surprising if they derived significant benefits from additional time, or experienced emotional costs as a result of reduced time with the partner. Instead, we found no effects of attachment avoidance. We note that our sample was, overall, relatively securely attached; it may be that we had an insufficient number of avoidantly attached individuals to adequately test for moderation. Alternatively, it is possible that the uncertainties associated with the pandemic were so extraordinary that even avoidantly attached individuals benefitted from the reassuring presence of the partner.

Indeed, social baseline theory (SBT; Beckes & Coan, 2011) would predict that when facing a threat, individuals will typically benefit from the presence of others, particularly those who are familiar and reliable, such as relationship partners. According to SBT, humans depend on or expect a default level of access to a social network in which they share goals and are interdependent with others. To the extent that this network is available, individuals will be less reactive to threats, because the risks associated with that threat are shared, and the burden or “load” of responding is spread across the larger social landscape (Coan & Maresh, 2014; Coan & Sbarra, 2015). The proximity of others thus allows for effective emotion regulation, whereas social isolation can lead to a decrease in the ability to regulate emotions (Diamond et al., 2008; Sbarra & Nietert, 2009). The large-scale isolation brought on by the pandemic may have made it more difficult for individuals to regulate their emotions, leading to greater stress. Indeed, numerous studies have now identified heightened stress and anxiety associated with COVID-19 (e.g., Barzilay et al., 2020; Jenkins et al., 2021; Robillard et al., 2020; Xiong et al., 2020). A reduction of time with a romantic partner may have increased stress, even for relatively avoidantly attached individuals, with subsequent costs to relationship quality.

We found no evidence that extra time spent with a partner increased stress or exacted costs on relationships; this finding runs counter to the “pressure cooker” narrative entertained by some media outlets, which suggested that couples forced to spend additional time together during trying times may have experienced problems in their relationship (Savage, 2020; Stroh, 2020). It is possible that, because we collected our data several months into the pandemic, at least some individuals who had experienced negative outcomes due to additional time together had already ended their relationships and thus were not included in our study. Further, such effects may have been limited to couples also experiencing other pressures, such as the difficulties of caring for young children while also working from home, or those managing financial problems. Alternatively, it may be that, for most individuals, the benefits of having a support figure close at hand during a crisis outweighed the minor irritations that arose from extra time together. Indeed, our findings are consistent with other studies suggesting that individuals who were locked down with their partners did not experience negative outcomes arising from the shared confinement and, if anything, benefitted from the extra time with the partner (e.g., Galdiolo et al., 2022; Günther-Bel et al., 2020). In future research, it will be important to assess whether there are indeed circumstances under which additional time with the partner has a negative impact on stress and relationship quality.

There are several limitations to the present research. Methodologically, we relied on single-item measures for some of our variables, including perceptions of time with friends and family; because such measures are unreliable, this may in part account for our lack of findings with respect to these variables. In addition, our conclusions are limited because we were forced to rely on participants’ memories of how much time they were spending with their partner prior to the pandemic. Past research, however, suggests that retrospective measures can be valuable in studying the impact of unforeseen events (e.g., Uttl et al., 2018), and that such measures can indeed be quite accurate (Howard, 2011; Lawson et al., 2020). Nevertheless, it will be important in future research to assess time spent together with more reliable and objective measures.

We also note that our conclusions are of necessity drawn from correlational methodology, and that our findings are thus open to alternative explanations. For example, it may be that individuals who were experiencing high levels of stress were choosing to spend less time with their partners. This seems unlikely; the pandemic, rather than stress, is a more obvious culprit in forcing couples to spend time apart, given that widespread public health measures had significantly limited individuals’ movements and socializing options. Conversely, it may be that individuals who are in higher quality relationships experienced less stress, which in turn led them to choose to spend more time with their partner. Given that this alternative model was significant only for commitment, and not relationship satisfaction, our model employing a change in time together as a predictor provides a more comprehensive and parsimonious explanation for the relationships among these variables. In addition, the theoretical rationale for a model in which relationship outcomes precedes stress is weaker than the well-established theoretical rationale, grounded in the literature on attachment, for a model in which the loss of time with a partner may result in stress (Hazan & Shaver, 1987), and in which stress predicts negative relationship outcomes (e.g., Bodenmann, 1997; Karney & Bradbury, 1995). Although it is likely that individuals who are dissatisfied in their relationships may as a result experience stress, there is less evidence that stress would be associated with a reduction in time spent with the partner. Indeed, past evidence suggests that individuals under stress are more likely to seek time with a partner than to avoid the partner’s company (Collins & Feeney, 2000; Feeney & Collins, 2019).

The present study provides novel evidence regarding how a change in time with the partner may be related to stress and relationship outcomes. Given that these changes were outside participants’ control, we note that this finding may be most generalizable to situations in which individuals do not choose how much time they are spending with the partner (as with military deployments). It may be that the impact of a change in time together is different when that change is chosen rather than forced upon one, as when an individual decides to move further away from the partner or elects to spend more time at work rather than with the partner. Under such conditions, a reduction in time spent together may be less stressful, given that individuals may believe they have the option to reverse this change.

This research has important implications for practitioners and researchers seeking to understand ongoing effects the pandemic may be having on relationships. Our study demonstrates that the stress of a reduction in time together is coming at a cost for relationships, whereas extra time together may reduce stress and benefit relationships. Future research should examine whether such changes are affecting other family members, including children. That is, if changes in time together are causing stress for couples, their children may also experience stress and other negative effects.

The COVID-19 pandemic affected nearly every aspect of individuals’ lives, as people around the world changed their work, social activities, and health-related behaviors. Without doubt, the upheavals associated with the pandemic have had implications for romantic relationships; indeed, past research suggests that relationship outcomes have suffered as a result of COVID-19 (Pieh et al., 2020; Pollard & Rogge, 2022). The present study suggests that individuals who experienced reduced time with their partner had higher stress levels, which in turn contributed to these negative relationship outcomes, even after 10 months. In the future, it will be important to better understand whether individuals’ experiences in the early stages of the pandemic will have ongoing effects on their relationships.

## Supplemental Material

Supplemental Material - Love under lockdown: How changes in time with partner impacted stress and relationship outcomes during the covid-19 pandemic Click here for additional data file. Supplemental Material for Love under lockdown: How changes in time with partner impacted stress and relationship outcomes during the covid-19 pandemic by Kaitlin Derbyshire, Sabrina Thai, Claire Midgley and Penelope Lockwood in Journal of Social and Personal Relationships

## Notes

1. The measure of quality time was included only at the second of two data collection points (T2), as will be discussed below.

2. Initially, we intended to focus our study on the impact of perceived time spent together on relationship outcomes (https://osf.io/4vyx2); however, given the growing amount of evidence regarding the impact of the pandemic on stress (e.g., Barzilay et al., 2020; Jenkins et al., 2021; Robillard et al., 2020; Xiong et al., 2020), we chose to examine stress as a mediator. We note that our original hypotheses regarding attachment as a moderator for analyses regarding relationship outcomes were not supported. Additional details on our findings regarding attachment are available in supplemental materials here: (https://osf.io/e43sj).

3. Due to a procedural error, 98 participants did not receive an attention check; these participants were included in the analyses, given that inattentive participants would likely introduce noise rather than bias in the data.

4. Eight of the 39 participants who were no longer in the same relationship were among those excluded for failing the attention check. Data from one participant whose partner had died was not included in analyses regarding relationship status as an outcome.

5. We also included a second measure of a change in time spent together, in which participants were asked to estimate how many hours on average they were spending per day with their partner, both currently and prior to the start of the pandemic. The pattern of results for hours with partner in the present, controlling for hours with partner pre-pandemic, was generally consistent with that obtained with the four-item perceptions of time measure, although some analyses did not reach significance. In the interest of concision, and because we deemed the four-item perceptions measure to be more reliable than the single-item hours measure, we report the regression and mediation analyses for the number of hours measure in supplemental materials: https://osf.io/e43sj.

6. Age, gender, and whether participants were cohabitating with partners during the pandemic did not moderate the effect of T1 perceived change in time spent with the partner on T1 stress, *t*s < 1.45, *p*s > .148.

7. We also considered the possibility that stress might serve as a moderator of the effect of time together on relationship outcomes rather than a mediator; specifically, individuals experiencing higher levels of stress might benefit more from an increase in time with the partner than would individuals experiencing less stress at both time points. T1 stress did not moderate the effect of T1 time together on relationship quality at T1 or T2, *t*s < 1.61, *p*s > .108. See Supplementary Materials (p. 18).

8. We also considered an alternative model addressing the possibility that individuals in more committed or satisfied relationships would choose to spend more time together during the pandemic, which in turn would reduce their level of stress. The indirect effect of T1 commitment on T1 stress through a change in time spent with partner at T1 was significant, *b* = −0.01, *SE =* 0.01, CI [-0.03, −0.001], but the indirect effect of T1 satisfaction on T1 stress through a change in time spent with partner at T1 was not significant, *b* = −0.01, *SE =* 0.005, CI [-0.02, 0.001]. In addition, we tested whether this model would account for T2 stress. The indirect effect of T1 commitment on T2 stress through T1 change in time with partner was significant, *b* = −0.02, *SE* = 0.01, CI [-0.04, −0.001]; however, the indirect effect of T1 satisfaction on T2 stress through T1 change in time with partner was not significant, *b* = −0.01, *SE* = 0.01, CI [-0.03, 0.0001].

9. This test indicates that the critical point (i.e., when the line plateaus) is not significantly different from 0.
