# Supplementary material for: Love under lockdown: How changes in time with partner impacted stress and relationship outcomes during the COVID-19 pandemic
Source: J Soc Pers Relat. 2023 Mar 9;40(9):2918–45. doi: 10.1177/02654075231162599 (PMC10009005; doi:10.1177/02654075231162599)
Supplement: Supplemental Material - Love under lockdown: How changes in time with partner impacted stress and relationship outcomes during the covid-19 pandemic [file sj-pdf-1-spr-10.1177_02654075231162599.pdf]

Love Under Lockdown: How Changes in Time with Partner Impacted Stress and Relationship Outcomes During the Covid-19 Pandemic: Online Supplement

Additional Analyses	Page
I. Moderation by Age, Gender, and Cohabitation During Pandemic	2
II. Moderation by Perceptions of COVID Conditions	2
III. Moderation of Time Together on Relationship Quality by Time with Friends and Family	4
IV. Moderation by Attachment at Time 1 and Time 2	5
V. Change in Time Spent Together (In Hours)	9
VI. Moderation by Stress (Alternative Model)	16
VII. Moderation by Time Together (Alternative Model)	17
VIII. Moderation of Time Together by Stress (Alternative Model)	18
IX. Additional Demographics Information About Time 2 Sample	19

.

The following analyses examine whether the association between time with partner and stress at Time 1 was moderated by other variables, such as demographic variables or COVID conditions.

Moderation by Age, Gender, and Cohabitation During the Pandemic

We tested whether the association between time spent with partner and stress was moderated by age, gender, and cohabitation during the pandemic in three separate models (i.e., one for each moderator). For age, we grand-mean centered age before entering it into the model. For gender, we effects coded gender (-1 = female; +1 = male) before entering it into the model. For these analyses, we excluded 5 participants who indicated they identified as nonbinary. For cohabitation during the pandemic, we also effects-coded cohabitation status (1 = Yes; -1 = No) before entering it into the model. Age, $b = -0.02$, $SE = 0.02$, $t(566) = -0.87$, $p = .383$, $r = .04$, gender, $b = 0.02$, $SE = 0.02$, $t(561) = 1.05$, $p = .293$, $r = .04$, and cohabitation during the pandemic, $b = 0.04$, $SE = 0.03$, $t(566) = 1.45$, $p = .148$, $r = .06$, did not moderate the association between time with partner and stress.

Moderation by Perceptions of COVID Conditions

We also examined whether perceptions of COVID restrictions at Time 1 and severity of COVID at Time 1 moderated the effect of change in time together at Time 1 on stress at Time 1. Neither perceptions of COVID restrictions, $b = 0.01$, $SE = 0.02$, $t(566) = 0.44$, $p = .659$, $r = .02$, nor perception of the severity of COVID, $b = 0.03$, $SE = 0.02$, $t(565) = 1.81$, $p = .071$, $r = .08$, moderated the effect of time together on stress.

This lack of moderation may be due to the limited variability in these measures. Indeed, 498 (87.52%) participants in our sample reported a response at or above the midpoint for how severe they perceived COVID to be in their region. Similarly, 552 (96.84%) participants in our

sample reported a response at or above the midpoint for COVID restrictions, suggesting that almost the entire sample was living in regions where restrictions were imposed. Moreover, 422 (74.04%) participants in our sample reported living in regions with many restrictions (i.e., indicated scores of 5 or 6 on our 0 to 6 scale).

Moderation of Time Together on Relationship Quality by Time with Friends and Family

We also examined whether the association between time spent with partner and relationship quality (i.e., satisfaction and commitment) at Time 1 was moderated by changes in time spent with family and friends at Time 1. For commitment, time with family, $b = 0.01$, $SE = 0.01$, $t(565) = 0.73$, $p = .468$, $r = .03$, and time with friends, $b = 0.01$, $SE = 0.02$, $t(566) = 0.31$, $p = .759$, $r =.01$, did not moderate the association between time with partner and commitment at Time 1. For satisfaction, time with family, $b = 0.02$, $SE = 0.02$, $t(565) = 1.01$, $p = .311$, $r = .04$, and time with friends, $b = -0.01$, $SE = 0.03$, $t(566) = -0.26$, $p = .791$, $r = .01$, did not moderate the association between time with partner and commitment at Time 1.

Moderation by Attachment at Time 1 and Time 2

We had pre-registered hypotheses regarding attachment anxiety and avoidance as possible moderators of the impact of a change in time spent together on relationship outcomes. In general, we expected that more avoidantly attached individuals would report more negative relationship outcomes when they experienced an increase in time spent together, and more anxiously attached individuals would report more positive relationship outcomes when they experienced an increase in time spent together. To test this possibility, we regressed each outcome (i.e., stress, commitment, and satisfaction at Time 1 and Time 2) on attachment anxiety (grand-mean centered), attachment avoidance (grand-mean centered), change in time with partner, the attachment anxiety by time with partner interaction, and the attachment avoidance by time with partner interaction.

Time 1

Commitment. We first examined whether participants' commitment ratings depended on attachment avoidance and the change in time spent with their partner during (vs. before) the COVID-19 pandemic. The main effect of attachment avoidance was significant, with more avoidance predicting lower commitment, b = -0.64, SE = 0.04, $t(564)$ = -16.76, p < .001, r = .58. In addition, there was a significant interaction between attachment avoidance and subjective time difference, controlling for both attachment anxiety and the attachment anxiety by subjective time difference interaction, b = 0.08, SE = .02, $t(564)$ = 3.93, p < .001, r = .16. At low levels of attachment avoidance (-1 SD), change in time with partner did not predict commitment, b = -0.02, SE = 0.03, $t(564)$ = -0.58, p = .563, r = .02. At high levels of attachment avoidance (+ 1 SD), more time with partner was associated with higher levels of commitment, b = 0.15, SE =

0.03, $t(564) = 5.14$, $p < .001$, $r = .21$. Neither the main effect of attachment anxiety nor the attachment anxiety by time spent with partner were significant, ts < 1.60, ps $> .110$.

Satisfaction. We next examined whether participants' satisfaction ratings depended on their levels of attachment avoidance or attachment anxiety and change in time spent with partner. The main effect of attachment avoidance was significant, with more attachment avoidance predicting lower satisfaction, $b = -0.75$, $SE = 0.04$, $t(564) = -17.44$, $p < .001$, $r = .59$. The main effect of attachment anxiety was also significant, with more attachment anxiety predicting lower satisfaction, $b = -0.27$, $SE = 0.04$, $t(564) = -6.21$, $p < .001$, $r = .25$. In addition, there was a significant interaction between attachment avoidance and subjective time difference, controlling for both attachment anxiety and the attachment anxiety by subjective time difference interaction, $b = 0.06$, $SE = 0.02$, $t(564) = 2.63$, $p = .009$, $r = .11$. At low levels of attachment avoidance (-1 SD), change in time with partner did not predict satisfaction, $b = -0.01$, $SE = 0.03$, $t(564) = -0.34$, $p = .736$, $r = .01$. At high levels of attachment avoidance, more time with partner was associated with higher levels of satisfaction, $b = 0.11$, $SE = 0.03$, $t(564) = 3.49$, $p = .001$, $r = .15$. The attachment anxiety by time spent together interaction was not significant, $b = -0.01$, $SE = 0.02$, $t(564) = -0.40$, $p = .691$, $r = .02$.

Stress. We then examined whether participants' stress level depended on time spent together moderated by attachment anxiety and attachment avoidance. The main effect of attachment anxiety was significant, with more attachment anxiety predicting greater stress, $b = 0.33$, $SE = 0.03$, $t(564) = 10.17$, $p < .001$, $r = .39$. The main effect of attachment avoidance was also significant, with more attachment avoidance predicting greater stress, $b = 0.07$, $SE = 0.03$, $t(564) = 2.11$, $p = .035$, $r = .09$. The main effect of time together, however, was no longer

significant, *b* = -0.02, *SE* = 0.02, *t*(564) = -1.33, *p* = .183, *r* = .06. No interactions were significant, *t*s < 0.76, *p*s > .446.

Time 2

Commitment. We next examined whether attachment anxiety and attachment avoidance (measured at Time 1) moderated the effects of a change in time spent together at Time 1 on outcomes at Time 2. The main effect of attachment avoidance was significant, with more avoidantly attached individuals reporting lower commitment, *b* = -0.42, *SE* = 0.05, *t*(353) = -8.46, *p* < .001, *r* = .41. Neither the main effect of attachment anxiety nor the attachment avoidance by time together or attachment anxiety by time together interactions were significant, all *t*s < 1.05, *p*s > .294.

Satisfaction. The main effect of attachment avoidance was significant, with more attachment avoidance predicting lower satisfaction, *b* = -0.48, *SE* = 0.06, *t*(353) = -7.81, *p* < .001, *r* = .38. The main effect of attachment anxiety was also significant, with more attachment anxiety predicting lower satisfaction, *b* = -0.16, *SE* = 0.06, *t*(353) = -2.60, *p* = .010, *r* = .14. No interactions were significant, *t*s < 0.58, *p*s > .562

Stress. The main effect of attachment anxiety was significant, with more anxiously attached individuals reporting more stress, *b* = 0.29, *SE* = 0.04, *t*(353) = 6.82, *p* < .001, *r* = .34. The attachment anxiety by time spent together interaction was also significant, *b* = -0.05, *SE* = 0.02, *t*(353) = -2.18, *p* = .030, *r* = .12. At low levels of attachment anxiety (-1 *SD*), more time together did not predict stress, *b* = -0.01, *SE* = 0.03, *t*(353) = -0.22, *p* = .827, *r* = .01. At high levels of attachment anxiety (+1 *SD*), more time together predicted lower stress, *b* = -0.11, *SE* = 0.03, *t*(353) = -3.34, *p* < .001, *r* = .17. Neither the main effect of attachment avoidance nor the attachment avoidance by time together interaction was significant, *t*s < 1.05, *p*s > .293.

In sum, we found no evidence for our preregistered hypotheses regarding the moderating impact of attachment style on the effect of a change in time spent together on relationship outcomes. That is, we found no evidence that avoidantly attached individuals would experience more negative relationship outcomes when spending more time with their partner, or that anxiously attached individuals would experience more positive relationship outcomes when spending more time with their partner.

Change in Time Spent Together (In Hours)

Participants indicated the average number of hours they spent with their partner each day before the pandemic began, and then the average number of hours they spent each day with their partner currently. This measure enabled us to assess the amount of time participants were spending daily with their partner during the pandemic, controlling for the amount of time they had spent with their partner daily before the pandemic started. We grand-mean centered both predictors. When testing for moderations, we also included the interaction term with amount of time participants were spending daily with their partner before the pandemic because we were interested in whether the change in time was moderated.

Thus, the model we were interested in testing is as follows:

$$Outcome = b_0 + b_1 Hours\ During + b_2 Hours\ Before + b_3 (Hours\ During - Hours\ Before)(Moderator)$$

This model expands to the following:

$$Outcome = b_0 + b_1 Hours\ During + b_2 Hours\ Before + b_3 (Hours\ During)(Moderator) - b_4 (Hours\ Before)(Moderator)$$

We chose not to constrain b_3 and b_4 to be equal in our model as this is an unnecessary constraint to impose on our model.

Time 1 Analyses

Change in Time Spent Together (in hours)

A paired *t*-test using the number of hours participants estimated spending with their partner before and during the pandemic revealed that participants reported spending significantly more hours with their partner during the pandemic, $t(569) = 4.53$, $p < .001$, Cohen's $d = 0.19$ (see Table S1 for means).

Stress

The number of hours spent together during the pandemic was not associated with stress, controlling for hours spent together during the pandemic, *b* = -0.06**,** *SE* = 0.04, *t*(567) = -1.35, *p* = .176, *r* = .06. Further, we found no evidence of moderation by whether participants were living together or apart during the pandemic, the amount of time they spent with family, or the degree of COVID-19 restrictions in their region, *t*s < 1.39, *p*s > .164.

The effect was, however, moderated by time they spend with friends, *b* = 0.08, *SE* = 0.03, *t*(564) = 2.48, *p* = .013, *r* = .10. For participants who spent less hours with their partner during the pandemic (i.e., 1 *SD* below the mean), more time with friends predicted less stress, *b*=-0.12, *SE*=0.04, *t*(564)=-2.65, *p*=.008, *r*=.11. In contrast, for participants who spent more hours with their partner during the pandemic (i.e., 1 *SD* above the mean), more time with friends was associated with less stress, *b* = 0.04, *SE* = 0.04, *t*(564) = 1.10, *p* = .270, *r* = .05.

This effect was also moderated by the perceived severity of COVID-19 in the region, *b*=0.10, *SE*=0.04, *t*(563)=2.36, *p*=.018, *r*=.10. For participants who perceived low levels of COVID-19 severity, more time with their partner predicted lower stress, *b* = -0.19, *SE* = 0.07, *t*(563) = -2.75, *p* = .006, *r* = .12. For participants who perceived greater severity of COVID-19, there was no effect of time with their partner on stress, *b* = 0.02, *SE* = 0.06, *t*(563) = 0.38, *p* = .703, *r* = .02.

Curvilinear Analyses. We also considered the possibility that a change in time spent together might have a curvilinear association with stress. That is, it may be that stress suffered when individuals were spending either too little or too much time with their partners as a result of the pandemic. To test this possibility, we tested for the curvilinear effect of the difference in time before and during the pandemic using the following equation:

$$Outcome = b_0 + b_1 Hours\ During + b_2 Hours\ Before + (Hours\ During - Hours\ Before)^2$$

The equation above expands to the following:

$$Outcome = b_0 + b_1 Hours\ During + b_2 Hours\ Before + b_3 (Hours\ During)(Hours\ Before) + b_4 Hours\ During^2 + b_5 Hours\ Before^2$$

Consequently, in our models testing for the curvilinear effect of the change in hours together, we included the linear effects of hours during and before the pandemic, their interaction, and the quadratic effects of hours during and before the pandemic. There was a significant curvilinear effect of time spent together during the pandemic on stress, *b* = 0.12, *SE* = 0.05, *t*(564) = 2.40, *p* = .017, *r* = .10. The positive sign suggests the curve has a concave upward shape. For people who were spending less time with their partner (i.e., 1 *SD* below the mean), spending more time with their partner was associated with less stress, *b* = -0.37, *SE* = 0.14, *t*(564) = -2.69, *p* = .007, *r* = .11. For people who were spending more time with their partner (i.e., 1 *SD* above the mean), however, spending time with their partner did not predict stress, *b* = 0.11, *SE* = 0.08, *t*(564) = 1.28, *p* = .203, *r* = .05. This pattern of results is consistent with the linear plateau model found for the subjective measures of change in time together reported in the main manuscript.

Commitment

The number of hours spent together during the pandemic was positively associated with commitment, controlling for hours spent together before the pandemic; that is, a relative increase in the number of hours spent with a partner was associated with greater commitment, *b* = 0.20, *SE* = 0.06, *t*(567) = 3.50, *p* < .001, *r* = .15. This effect was not moderated by whether couples

were cohabiting, whether they had children living in the home, participants' gender, the change in time spent during the pandemic with friends and family, their perceptions of the severity of COVID-19 in the region in which they were living, or the degree of COVID-19 restrictions in the region in which they live, *t*s < 1.96, *p*s > .051.

Curvilinear Analyses. We also considered the possibility that a change in time spent together might have a curvilinear association with commitment. That is, it may be that commitment suffered when individuals were spending either too little or too much time with their partners as a result of the pandemic. To test this possibility, we used the model specified above for stress. The curvilinear effect of time spent together during the pandemic on commitment was not significant, $b = -0.07$, $SE = 0.06$, $t(564) = -1.05$, $p = .294$, $r = .04$.

Satisfaction

The number of hours spent together during the pandemic was positively associated with satisfaction, controlling for hours spent together before the pandemic; that is, a relative increase in the number of hours spent with the partner was associated with greater satisfaction, $b = 0.15$, $SE = 0.07$, $t(567) = 2.09$, $p = .037$, $r = .09$. This effect was not moderated by whether couples were cohabiting, whether they had children living in the home, participants' gender, the change in time spent during the pandemic with friends and family, their perceptions of the severity of COVID-19 in the region in which they were living, or the degree of COVID-19 restrictions in the region in which they live, *t*s < 1.93, *p*s > .055.

Curvilinear Analyses. We also considered the possibility that a change in time spent together might have a curvilinear association with satisfaction. To test this possibility, we used the same model described above for stress. There was no significant curvilinear effect for time during the pandemic on satisfaction, $b = 0.003$, $SE = 0.08$, $t(564) = 0.03$, $p = .972$, $r = .00$.

Mediation Analyses

We next tested whether stress would mediate the effect of hours per day spent with partner during the pandemic on our outcome variables, controlling for hours per day spent with partner prior to the pandemic. The indirect effect of hours spent with partner on commitment, controlling for hours spent with partner prior to the pandemic, was not significant, b = 0.01, SE = 0.01, 95% CI [-0.01, 0.03]. Similarly, the indirect effect of hours spent with partner on satisfaction, controlling for hours spent with partner prior to the pandemic, was not significant, b = 0.03, SE = 0.02, 95% CI [-0.01, 0.07]. It is likely that the curvilinear association between time together during the pandemic and stress made it more difficult for us to detect an indirect effect.

Time 2 Analyses

As was the case at Time 1, participants reported spending more hours per day with their partner than they had before the pandemic (See Table S1). Indeed, a paired *t*-test using the number of hours participants estimated spending with their partner before and during the pandemic revealed that participants reported spending significantly more hours with their partner during the pandemic at Time 2 than before the pandemic, $t(358) = 2.69$, $p = .007$, *Cohen's d* = 0.14.

Table S1

Descriptive Data for Key Variables

	Participants with Data at T1 (*n* = 570)		Participants with Data at T1 and T2 (*n* =359)						
	**T1**		**T1**		**T2**				
Variable	*M*	*SD*	*M*	*SD*	*M*	*SD*	*t*	*p*	*d*
Perceived Change in Time Spent with Partner (+3 to -3)	0.28	1.70	0.22	2.01	0.18	1.67	0.33	.742	.02
Anxious attachment (0 to 6)	1.97	1.26							
Avoidant attachment (0 to 6)	1.56	1.02							
Hours Spent with Partner Currently (0-24)	10.18	8.35	10.63	8.37	9.10	6.80	4.01	<.001	.21
Hours Spent with Partner Pre-pandemic (0-24)	8.99	5.80	9.34	5.99	8.36	5.35	3.86	<.001	.20
Commitment (0 to 6)	5.20	1.05	5.32	0.89	5.21	0.92	2.72	.007	*.14*
Satisfaction (0 to 6)	4.50	1.29	4.59	1.23	4.52	1.17	1.31	.192	*.07*
Stress (0 to 4)	2.22	0.81	2.22	0.79	2.03	0.79	4.58	< .001	*.24*
COVID-19 Severity (0 to 6)	4.31	1.44	4.29	1.44	3.38	1.40	10.06	< .001	*.53*
Degree of COVID-19 Restrictions (0 to 6)	5.02	1.14	5.04	1.12	3.03	1.28	24.77	< .001	*1.31*

Note. *t*'s represent paired *t*-tests and *d*'s are Cohen's *d* effect size estimates. To ensure a more concise follow-up questionnaire, participants at Time 2 completed only one item assessing a change in time spent together ("I currently spent ___ time with my partner compared to before the pandemic started"). For comparison purposes, the *M* and *SD* for this item only, rather than the mean of the four-item composite, is provided here for Time 1. Attachment anxiety and avoidance were measured at Time 1 only.

We next examined whether participants' estimates of hours spent together on average per day at Time 1 predicted their outcomes at Time 2, after controlling for their estimates of time spent together before the pandemic.

Stress

The estimated number of hours spent together during the pandemic at Time 1, controlling for the Time 1 estimate of hours spent together before the pandemic, significantly predicted stress at Time 2, $b = -0.16$, $SE = 0.06$, $t(356) = -2.76$, $p = .006$, $r = .14$; participants who were spending more hours together at Time 1 reported lower stress at Time 2.

Commitment

The estimated number of hours spent together during the pandemic at Time 1, controlling for the Time 1 estimate of hours spent together before the pandemic, did not predict commitment at Time 2, $b = 0.09$, $SE = 0.07$, $t(356) = 1.30$, $p = .194$, $r = .07$.

Satisfaction

The estimated number of hours spent together during the pandemic at Time 1, controlling for the Time 1 estimate of hours spent together before the pandemic, did not predict satisfaction at Time 2, $b = 0.06$, $SE = 0.09$, $t(356) = 0.72$, $p = .473$, $r = .04$.

Moderation by Stress (Alternative Model)

We also considered the possibility that some couples may be more vulnerable to the stress experienced during the pandemic. That is, individuals with lower satisfaction at Time 1 or lower commitment at Time 1 were more likely to experience declines in satisfaction or commitment at Time 2 if they also experienced high levels of stress. To test this, we conducted four moderation analyses. In these models, all predictors were grand-mean centered. In the first model, we regressed Time 2 satisfaction on Time 1 satisfaction, Time 1 stress, and the Time 1 satisfaction by Time 1 stress interaction. This model revealed that there was no significant interaction between Time 1 satisfaction and Time 1 stress, $b = -0.06$, $SE = 0.05$, $t(355) = -1.10$, $p = .270$, $r = .06$. In the second model, we regressed Time 2 commitment on Time 1 commitment, Time 1 stress, and the T1 commitment by T1 stress interaction. This model revealed that there was no significant interaction between Time 1 commitment and Time 1 stress, $b = -0.08$, $SE = 0.06$, $t(355) = -1.25$, $p = .210$, $r = .07$. In our third model, we regressed Time 2 satisfaction on Time 1 satisfaction, Time 2 stress, and the Time 1 satisfaction by Time 2 stress interaction. This model revealed that there was no significant interaction between Time 1 satisfaction and Time 2 stress, $b = -0.03$, $SE = 0.06$, $t(355) = -0.41$, $p = .679$, $r = .02$. In the fourth model, we regressed Time 2 commitment on Time 1 commitment, Time 2 stress, and the T1 commitment by T2 stress interaction. This model revealed that there was no significant interaction between Time 1 commitment and Time 2 stress, $b = -0.03$, $SE = 0.06$, $t(355) = -0.53$, $p = .564$, $r = .03$. Given that our sample consisted of primarily securely attached individuals, it is possible that the number of participants who were vulnerable in this sample was too small for us to detect an effect if it did exist.

Moderation by Time Together (Alternative Model)

We also considered another model in which couples who were unhappy prior to the pandemic and who were forced to spend time together due to the pandemic may experience higher levels of stress. We tested this possibility in two separate models. In the first model, we regressed T1 stress on T1 satisfaction, T1 time together, and their interaction. There was no significant interaction in this model, $b = 0.01$, $SE = 0.01$, $t(566) = 0.72$, $p = .470$, $r = .03$. In the second model, we regressed T1 stress on T1 commitment, T1 time together, and their interaction. There was also no significant interaction in this model, $b = 0.004$, $SE = 0.02$, $t(566) = 0.23$, $p = .820$, $r = .01$.

Given that our sample consisted of primarily securely attached individuals, it is possible that there were not enough individuals who were unhappy with their relationship in our sample to detect this effect if it did exist.

Moderation of Time Together by Stress (Alternative Model)

We also considered the possibility that the association between time together and relationship outcomes (i.e., satisfaction and commitment at Time 1 and Time 2). To test this model, we regressed the relationship outcome on Time 1 time together, Time 1 stress (grand-mean centered), and their interaction. For Time 1 satisfaction, $b = 0.06$, $SE = 0.04$, $t(566) = 1.61$, $p = .108$, $r = .07$, and commitment, $b = 0.02$, $SE = 0.03$, $t(566) = 0.66$, $p = .507$, $r = .03$, stress did not moderate the effect of time together. For Time 2 satisfaction, $b = 0.03$, $SE = 0.05$, $t(411) = 0.53$, $p = .599$, $r = .03$, and commitment, $b = 0.05$, $SE = 0.04$, $t(411) = 1.05$, $p = .296$, $r = .05$, stress did not moderate the effect of time together.

Additional Demographics Information About Time 2 Sample

Of the 570 participants included in the analyses for T1, 417 completed the second survey. We excluded 39 participants who were single, widowed, or in a different relationship at T2, and 26 who did not pass an attention check. In total, our analyses included 359 participants who provided usable data at both time points (182 female, 174 male, 3 nonbinary; M_{age}=28.77, *SD*=8.85, range=18 to 63; $M_{relationship\ length}$=79.42 months, *SD*=77.12, range=12 to 495, *Mdn*=50.00). Of these participants, 296 identified as being heterosexual, 9 as being gay or lesbian, 30 as bisexual, 5 as queer, 6 as questioning, 1 chose not to answer, and 4 chose other (e.g., sapiosexual, pansexual). Three hundred and three participants reported living in Europe, 39 in North America, 4 in the Middle East, 2 in Asia/Pacific, 5 in Africa, 4 in Latin America, and 2 chose not to respond. Three hundred and three identified as White, 6 as African, 15 as South American, 3 as Middle Eastern, 1 as South Asian, 2 as Southeast Asian, 3 as East Asian, 20 as other, and 6 as multiracial. Sixty-seven participants had completed high school, 33 had completed some college, 75 had completed some university, 7 had completed an Associate degree, 114 had completed a Bachelor’s degree, and 63 had completed a Postgraduate degree. Our dataset examining relationship status as an outcome included 389 participants (201 female, 185 male, 3 nonbinary; M_{age}=28.30, *SD*=8.71, range=18 to 63, *Mdn*= 26.00).
